# Fine-tuning the use of a skin prick test device

**DOI:** 10.1016/j.waojou.2020.100122

**Published:** 2020-05-08

**Authors:** Melike Kahveci, Erdem Karabulut, Ozge Soyer, Umit Murat Sahiner, Betul Buyuktiryaki, Bulent Enis Sekerel

**Affiliations:** a Department of Pediatric Allergy, Hacettepe University Faculty of Medicine, 06100, Ankara, Turkey; b Department of and Biostatistics, Hacettepe University Faculty of Medicine, 06100, Ankara, Turkey

**Keywords:** Acceptability, Lancet, Oryum, Reproducibility, Sensitivity, Skin prick test, Specificity, Technique

## Abstract

**Background:**

Skin prick tests (SPTs) are the gold standard for the diagnosis of IgE-mediated allergic diseases. Newly introduced devices have different results in performance. This study aimed to provide data for sensitivity, reproducibility, and acceptability of a new SPT device by using different techniques.

**Methods:**

The study was conducted in 4 sections. Different application techniques were evaluated. In the first section, a drop of histamine/saline was put by vial (V). In the second section, it was taken from a well via the test device (W). ALK® Lancet served as a reference in both sections. The techniques were as follows; 1) apply vertical pressure (VP/WP), 2) apply vertical pressure and rotate 90° clockwise (VC/WC), 3) apply vertical pressure and rotate 90° clockwise and then counter-clockwise (VCC/WCC). Pain assessment was performed by using the Wong-Baker FACES Pain Rating Scale. Different histamine concentrations were transferred from the well by the Oryum device and applied as WC in section 3. Lancet and Oryum-WP were compared in terms of time and allergen adequacy in section 4.

**Results:**

In the first section the sensitivity of all techniques for Oryum and lancet were 100%. The false positivity of Oryum-VP, WP and lancet were found 0%. The Oryum-VP technique was found the best for intrapatient coefficient of variation (CV) (10.72%) (p < 0.001). The interpatient CV was similar in the Oryum-VP, VC, VCC and lancet techniques and was different from the Oryum-WP (p < 0.001). In the second section, all Oryum techniques yielded high sensitivities (100%). False-positive results were obtained more in Oryum-WC and WCC. Oryum-WP technique had the lowest pain score. In the 3rd section, the high positive correlation between histamine concentrations was observed (r = 0.731). In terms of time and allergen adequacy, Oryum-WP was superior to the lancet.

**Conclusion:**

Oryum-VP and WP techniques are reliable, tolerable and comparable with the lancet technique.

## Introduction

Epidermal skin prick testing (SPT) is one of the essential diagnostic tools used to confirm immunoglobulin E (IgE)-mediated allergic diseases. It is minimally invasive, inexpensive and results are available immediately.^1^ Although the SPT is the gold standard for the diagnosis of IgE-mediated allergic diseases, there has been no consistency in the techniques and devices used for these tests. The size of the wheal is influenced not only by the severity of the hypersensitivity but also various factors including the type of puncturing device, method used for puncturing, and skills of the tester. The situation is further being complicated by the introduction of new skin test devices by different manufacturers. In previous studies that compared the devices for skin, testing has revealed significant variability in the size of reaction to histamine, allergen extract, or saline.^2^, ^3^, ^4^, ^5^ Because skin test results help to guide therapeutics, test device reliability is important to minimize false-negative and false-positive results. Therefore, the device to be used in clinics should be validated by using negative and positive controls, and appropriate application methods are required.^6^ Comparison studies examining the performance of different devices provide useful and important information for physicians.

The studies concerning SPT devices have been conducted mainly in adults so limited data are available on children. The aim of this study was to provide data for sensitivity, reproducibility, and acceptability of a new SPT device by using different techniques.

## Methods

### Study design

This prospective partially blind study consisted of 3 complementary sections to test the sensitivity, reproducibility, and acceptability of a commercially available device (Oryum®, Gaziantep, Turkey) with different techniques on children. Skin testing was performed on the backs of the patients with histamine (10 mg/mL; ALK®) and saline (Polifleks®) during a single session with a spacing of at least 20 mm between each test site. Histamine or saline was applied by different techniques in two sections. In the first section, a drop of histamine/saline was put by vial (V). In the second section, it was taken from a well via the test device (W). Lancet (ALK®, Horsholm, Denmark) device served as a reference in both sections.

In section 1, two hypotheses were tested whether different techniques give varying results and whether allergen can be transferred to the skin via the device. Oryum® device was used in each patient by the following methods: 1) drop and apply vertical pressure (Oryum-VP); 2) drop, apply vertical pressure and rotate 90° clockwise (Oryum-VC); 3) drop, apply vertical pressure and rotate 90° clockwise and then counter-clockwise (Oryum-VCC); 4) dip the device in a well containing histamine/saline, transfer the solution through the device and apply vertical pressure (Oryum-WP).

**Fig. 1:**
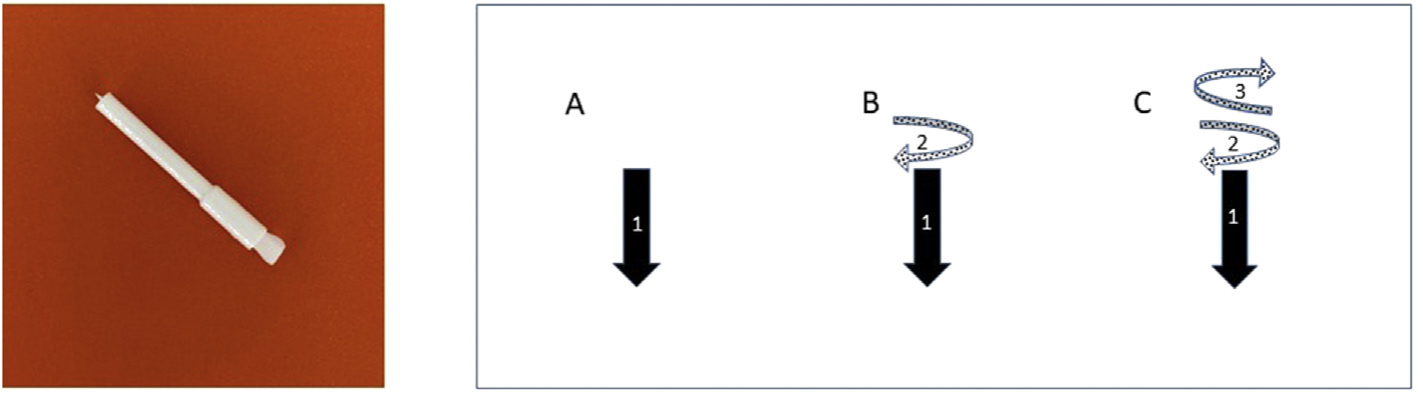
A picture of the ORYUM® device. A) Vertical pressure B) Vertical pressure and rotate 90° clockwise C) Vertical pressure and rotate 90° clockwise and then counter-clockwise

In section 2, the hypothesis of whether the device transferred histamine/saline gives different results along with varying techniques. Histamine/saline were taken from a well by Oryum device and applied by the following methods: 1) take and apply vertical pressure (Oryum-WP); 2) take, apply vertical pressure and rotate 90° clockwise (Oryum-WC); 3) take, apply vertical pressure and rotate 90° clockwise and then counter-clockwise (Oryum-WCC). A picture of the device was added, and different techniques used were shown with a diagram in Fig. 1.

Each Oryum technique was repeated 4 times with histamine and saline for all patients in sections 1 and 2, 1 and 2 to calculate the mean of wheal and flare diameter. Lancet device was applied 4 times with histamine and saline according to the techniques recommended by representatives of the manufacturer.^7^ The lancet means of wheal and flare diameter were found using 4 results. The total number of skin pricks for an individual subject was 40 for section 1 and 32 for section 2 of the study.

Pain assessment was performed by using the Wong-Baker FACES Pain Rating Scale immediately after the application of each test device technique. Pain scale ranged from 0 to 10, with 0 having no pain and 10 having the worst pain imaginable.^8^

In section 3, the hypothesis was whether histamine causes reactions parallel to its concentrations (1/1, 1/10, 1/100, 1/1000) if histamine is transferred from the well by the Oryum® device and applied as WP. Different histamine concentrations were applied once for each patient.

In section 4, the time for performing a set of 12 pricks was monitored on 5 patients, and the number of the pricks was measured using a 0.4 ml antigen by lancet and Oryum-WP techniques for 5 different times.

A single trained technician who performed both device applications was blind to the content of the applied solutions, either histamine or saline but not blind to the device due to the different appearances of the devices. Another trained technician measuring the wheals and flares was blind to the solution used and techniques. Before the study, the technician who performed the applications underwent an evaluation and a "coefficient of variation" (CV) of <20% was attained. The wheal and flare reaction diameters were recorded at 15 min by obtaining the longest and orthogonal diameters. The mean diameter was used for analysis.

### Study population

Subjects aged between 6 and 16 years with or without atopy were enrolled. The diagnosis of the patients was mild allergic rhinitis and/or asthma and/or atopic dermatitis. In the first section of the study 20 patients, in the 2nd section, 21 patients, and the 3rd section 16 patients, were included. Subjects were excluded if they had a systemic disease, dermatographism, severe atopic dermatitis, or asthma, or were taking antidepressants. Antihistamines were withheld for 1 week before the study. This study was approved by the local ethics committee (KA-180064) of the Hacettepe University Faculty of Medicine, and informed consent was obtained from both patients and their parents.

### Statistical analysis

Instruments and the techniques used in the tests were evaluated in terms of sensitivity, repeatability, and acceptability. According to the literature, the standard deviation between different techniques and devices was calculated as 2 mm.^9^ Since 5 different techniques were tested on 2 different devices, a total of 10 pairs of comparisons were possible. A sample size of 18 subjects was determined to have 80% power to detect a 2 mm diameter difference in the wheal reaction. The sensitivity of the techniques was calculated utilizing a positive threshold of 3 mm for the mean diameter of each wheal and found as equal to the ratio between the number of true positive tests and the sum of true positive and false negative ones. To determine the reproducibility of the results in the same patient and the results of the same technique from one patient to another, intrapatient and interpatient CV were calculated, respectively. The CV was noted as the standard deviation divided by the average of the mean diameters. To analyze the sensitivity and specificity, a true positive result was acknowledged as a histamine wheal of ≥3 mm, and a negative result was a 0.9% saline wheal of <3 mm. Since histamine measurements were normally distributed (the Shapiro Wilk test), differences in wheal diameter between techniques and devices were measured by repeated ANOVA testing. Pain scores were calculated by the Friedman test followed by the Dunn post-tests.

### Availability of data and materials

The datasets analyzed during the current study are available from the corresponding author on reasonable request. Informed consent was obtained from both patients and their parents.

## Results

### Section 1

Twenty patients with a median [interquartile range (IQR)] age of 10.6 (9.9–13.4) years participated in this section of the study. The sensitivity and specificity of the techniques along with the size of the reactions were summarized in Table 1.

**Table 1:** Measurements of different skin prick test techniques (Section 1).

	Histamine wheal, mean ± SD^a^	Histamine flare, mean ± SD^a^	CV % İntrapatient	CV % İnterpatient	Sensitivity % (95% CI)	Specificity % (95% CI)	Pain median (IQR)
Oryum-VP	5.25 ± 0.65^a^	21.84 ± 4.59^a^	10.72	27.16	100 (80–100)	100 (80–100)	4 (2–5.5)^a^
Oryum-VC	7.85 ± 1.19^b^	33.37 ± 3.6^b^	14.43	29.65	100 (80–100)	95 (73.1–99.7)	4 (4–6)^ab^
Oryum-VCC	7.86 ± 1.33^b^	31.93 ± 4.1^b^	12.32	29.36	100 (80–100)	95 (73.1–99.7)	5 (4–8)^b^
Oryum-WP	4.22 ± 0.89^c^	18.99 ± 4.5^c^	24.31	37.26	100 (80–100)	100 (80–100)	4 (2–6)^ab^
Lancet	5.45 ± 0.85^a^	24.85 ± 5.42^a^	11.99	28.88	100 (80–100)	100 (80–100)	5 (2.5–8)^ab^
P	<0.001	<0.001					0.008

^a, b, c^: different letters in the same column represent the statistically significant difference (p < 0.05).

a Values for wheal and flare expressed in millimeters

#### Wheal and flare responses

**Fig. 2:**
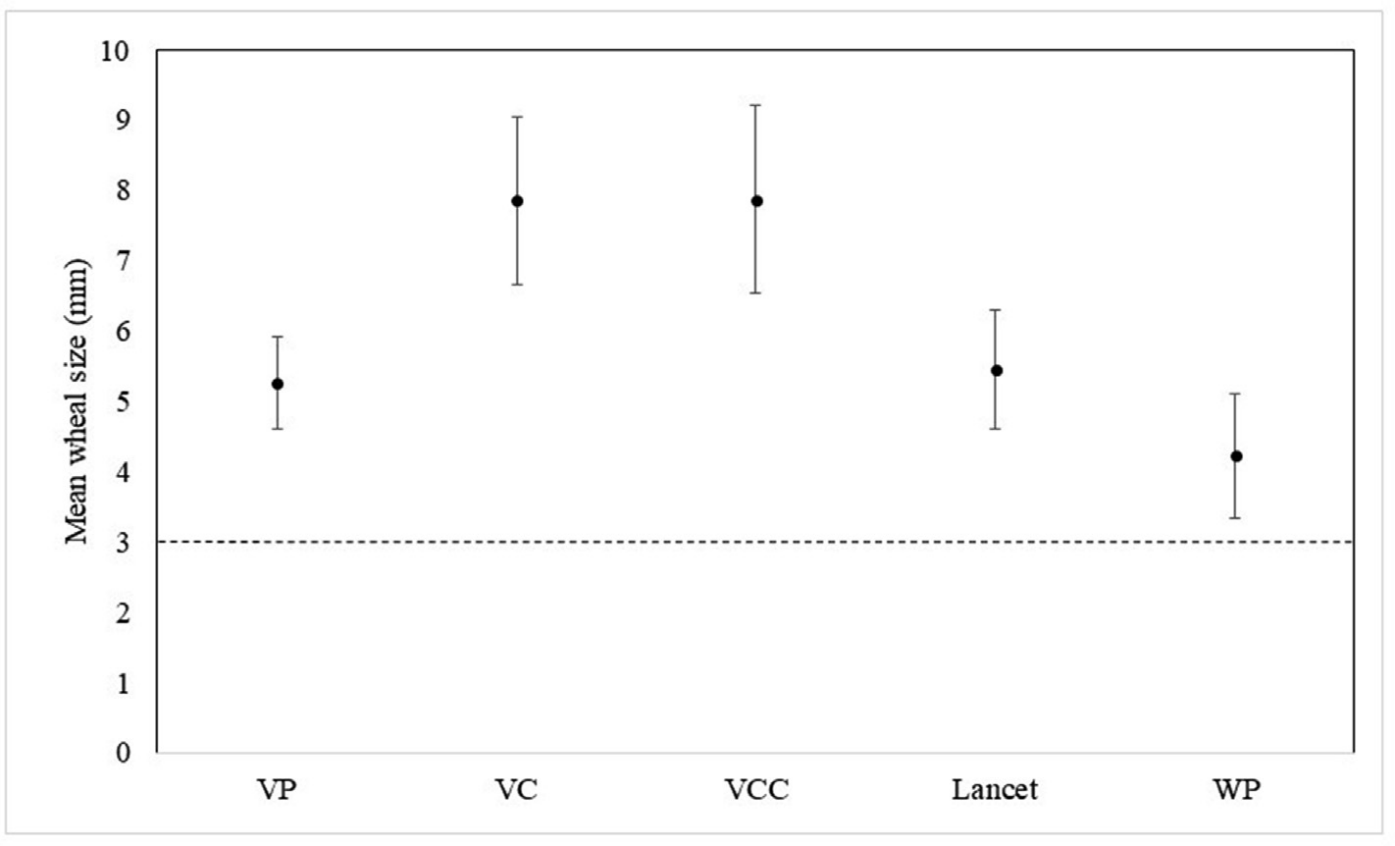
Mean wheal sizes between different techniques (section1). Error bars represent standard deviation

The mean [standard deviation (±SD)] diameter of wheals was ranged from 4.22 ± 0.89 mm to 7.86 ± 1.33 (p < 0.001) and flares were ranged from 18.99 ± 4.50 to 13.37 ± 3.62 (p < 0.001) (Fig. 2). The smallest wheal size was obtained by the Oryum-WP technique and larger wheal sizes were observed in Oryum-VC and Oryum-VCC techniques. The sizes of a histamine-induced wheal with Oryum-VP were not statistically different from the one achieved with a lancet however, Oryum-VC and VCC resulted in larger wheal sizes compared to the lancet. The rate of false negatives in the Oryum-VP, VC, VCC and WP techniques and lancet were 0%. The false positivity of Oryum-VC and VCC were 5% while Oryum-VP, WP and lancet were found 0%. The sensitivity of all techniques for Oryum and lancet was 100%, and there were no differences between these techniques.

#### Reproducibility of SPTs

The Oryum-VP technique was found to be the best for intrapatient CV comparison, followed by lancet and Oryum-VCC techniques (10.72%, 11.99%, and 12.32% respectively) (p < 0.001). The interpatient CV was similar in the Oryum-VP, Oryum-VC, Oryum-VCC, and lancet techniques and was different from the Oryum-WP technique (p < 0.001) (Table 1).

#### Pain scores

The pain score of the Oryum-VP technique was lowest. In the binary comparison between the techniques, Oryum-VP and Oryum-WCC pain scores were found different (p = 0.008), while other comparisons were similar (Table 1).

### Section 2

Twenty-one patients with a median (IQR) age of 10.47 (9.35–12.64) years participated in this section of the study. The sensitivity and specificity of the techniques along with the size of the reactions were summarized in Table 2.

**Table 2:** Measurements of different skin prick test techniques (Section 2).

	Histamine wheal, mean ± SD	Histamine flare, mean ± SD	CV % İntrapatient	CV % interpatient	Sensitivity % (95% CI)	Specificity % (95% CI)	Pain median (IQR)
Oryum-WP	4.61 ± 0.79^a^	18.79 ± 4.94^a^	12.66	21.15	100 (80.8–100)	95.2 (74.1–99.8)	2 (2–4)^a^
Oryum-WC	7.24 ± 1.73^b^	28.56 ± 5.5^b^	17.60	30.06	100 (80.8–100)	85.7 (62.6–96.2)	4 (2–4)^a^
Oryum-WCC	8.17 ± 1.70^c^	31.28 ± 6.44^c^	13.78	24.95	100 (80.8–100)	76.2 (52.5–90.9)	6 (2–7)^b^
Lancet	5.30 ± 0.67^d^	22.61 ± 5.1^d^	12.77	17.42	100 (80.8–100)	95.2 (74.1–99.8)	6 (2–8)^b^
P	<0.001	<0.001					<0.001

∗Values for wheal and flare expressed in millimeters.

^a, b, c, d^: different letters in the same column represent the statistically significant difference (p < 0.05)

#### Wheal and flare responses

**Fig. 3:**
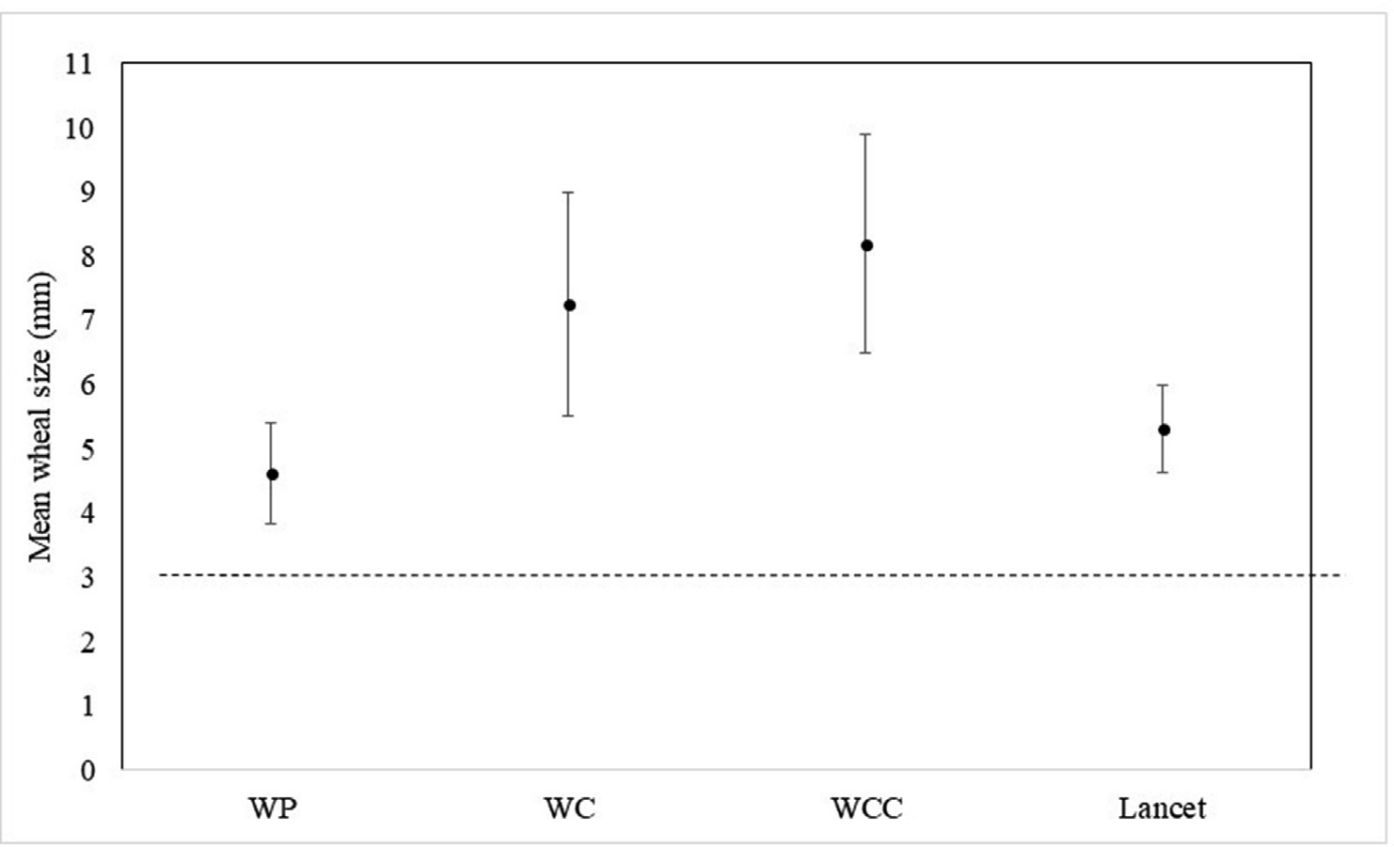
Mean wheal sizes between different techniques (section2). Error bars represent standard deviation

The mean (±SD) diameter of wheals was ranged from 4.61 ± 0.79 mm to 8.17 ± 1.70 (p < 0.001) and flares were ranged from 18.79 ± 4.94 to 31.28 ± 6.44 (p < 0.001) (Fig. 3). The smallest wheal size was obtained by the Oryum-WP technique, and the largest wheal size was observed in the Oryum-WCC technique. The approximate wheal size with lancet was observed in the Oryum WP technique. The rate of false negatives in the Oryum-WP, WC, WCC, and lancet was 0%. The false positivity of Oryum-WP and lancet were found similar (4.8%) (Table 2).

The sensitivity of all techniques for Oryum and lancet was 100%, and there were no differences between these techniques.

#### Reproducibility of SPTs

All techniques were found similar for intrapatient CV comparison (p = 0.202). In interpatient CV comparisons, Oryum-WP and lancet were found similar (21.15% and 17.42%, respectively), while the other techniques were different (p = 0.001).

#### Pain scores

The Oryum-WP technique had the lowest pain score. The pain scores of the lancet and Oryum-WCC techniques were found similar and they had higher pain scores (p < 0.001).

### Section 3

**Fig. 4:**
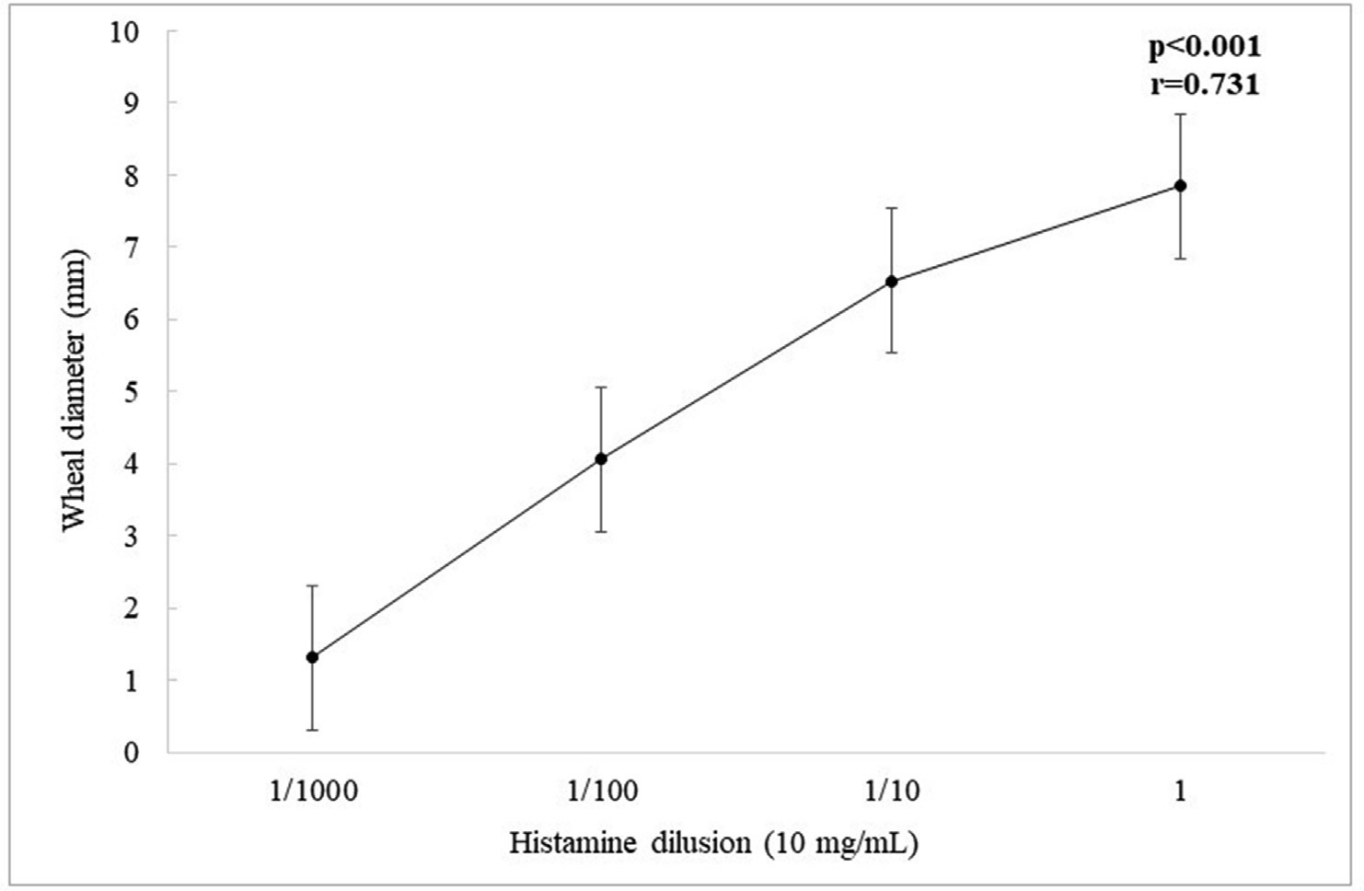
The mean diameters of wheals with different histamine dilutions

Sixteen patients with a median (IQR) age of 10.0 (8.6–12.2) years participated in this section of the study. Different histamine dilutions (1/1, 1/10, 1/100, 1/1000) were applied and the mean diameters of wheals were 7.84 ± 2.29, 6.53 ± 1.76, 4.06 ± 1.09, 1.31 ± 1.22 respectively. There was a high positive correlation between concentrations (p < 0.0001, r = 0.731) (Fig. 4).

### Section 4

Lancet and Oryum-WP were compared in terms of time and allergen adequacy. The time for 12 pricks for the standard panel was 119 ± 3 s by lancet and 54 ± 3 s by Oryum-WP (p < 0.001). Total pricks count with 0.4 ml allergen extract was 57 ± 5 with lancet and 86 ± 8 with Oryum-WP (p = 0,001) (Table 3).

**Table 3:** Comparison of 2 techniques in terms of time and allergen adequecy (n = 5)

	Lancet	Oryum-WP	P
Time, s (for 12 pricks in standard panel)	119 ± 3	54 ± 3	< *0.001*
Number of pricks (0.4 ml allergen extract)	57 ± 5	86 ± 8	*0.001*

## Discussion

This study showed that the Oryum VP method is as reliable as the lancet method. Although Oryum WP produces slightly less wheal size, its performance in terms of sensitivity and reliability is comparable to the lancet. Additionally, SPT with WP is faster, has more acceptable pain scores and causes less time and allergen use.

Skin prick testing remains the most often used tool in allergy practice. Over the years, different devices have been marketed to perform SPT; however, there are few studies in the literature comparing the performance of various devices in terms of their performance and usefulness.^3^^,^^4^ Therefore we designed this study to evaluate the sensitivity, reproducibility, and acceptability of the Oryum device, a new SPT device in the market. In the first section of our study, acceptable results were observed with all techniques however excellent sensitivity, and good specificity comparable to lancet was obtained with Oryum-VP and WP. Intrapatient and interpatient CV values were superior in Oryum-VP and lancet.

The second section of the study was conducted to test if the low wheal diameter of the Oryum-WP technique could be improved with the addition of rotation. Oryum-WC and WCC techniques had increased wheal diameters. In this case, the sensitivity was the same but the false positivity rate was found high. Although the wheal diameter of Oryum-WP was smaller, the sensitivity was 100%. Besides, intrapatient and interpatient CV results comparable to lancet were also detected in Oryum-WP. The results obtained from the second section of the study showed that the application technique, rather than the solution technique method, is important for getting false-positive results.

In the literature, it is observed that the sensitivity of the devices varies depending on the technique and device type. In a study investigating 4 devices including intravenous needle, ALK lancet, Stallergenes prick lancet, Stallerpoint®, Stallerpoint® 90, the sensitivities were found 100%, 96%, 98%, 20%, and 57%, respectively.^10^ In another study reported that sensitivity changed between 28.8% and 98.8%.^11^ Moreover, unlike other studies published on this topic, we found 100% sensitivity with all Oryum device techniques. The different sensitivities between these studies may depend on the factors related to the performance of the technicians and the device.

The coefficient of variations of the devices and techniques were calculated to assess precision and reproducibility. In our previous study, we evaluated the sensitivity, reproducibility, and acceptability of commercially available SPT devices (Stallerpoint, Antony, France and ALK lancet) found that the lancet and Stallerpoint-VC achieved the best results, whereas Stallerpoint-WP elicited the highest intrapatient and interpatient variability.^11^ In another study comparing 4 instruments (the 23G intravenous needle, the ALK Lancet, the Stallergenes Prick Lancet, and the Stallerpoint) by using the techniques in accordance with the manufacturer recommendations intrapatient reproducibility was detected as 16.2%, 14.6%, 15.0%, 97.1% and 18.1% (second technique for Stallerpoint) respectively. It was concluded that the Stallerpoint's (standard) reproducibility appeared to be insufficient for clinical usefulness (intrapatient and interpatient CV of 97.1% and 79.9%, respectively).^10^ In our study, Oryum-VP and WP techniques appeared reliable compared with the lancet technique.

We demonstrated that there were significant differences in the pain produced by the different SPT techniques. The more acceptable techniques due to pain scores were found as Oryum-VP and WP in our study. Higher pain scores were observed with the lancet, Oryum-VCC, and WCC techniques. In literature, the studies about the optimizing devices in SPT were all on adults, and the median pain scores were found low as 1.0–4.0.^4^^,^^9^^,^^10^ The only study on children, Buyuktiryaki et al. found the pain scores median 2.0–4.0, and the more painful techniques were VCC and WCC that were similar in our study.^11^

In the third section of the study, we applied different histamine dilutions for assessing the linearity of the Oryum® device. The functionality of the device even at very low concentrations was demonstrated.

The Oryum-WP technique had some advantages over the lancet. First, it reduces the cost of allergen. The tests could be applied to more patients with the same amount of allergen extract (p = 0.001). The technique of taking allergen from well with the device is a timesaving procedure. The drop-and-apply method with the lancet technique took approximately double-time compared with Oryum-WP (p < 0.001). Similarly, in the study byBuyuktiryaki et al., taking from well with device technique was found to be advantageous in terms of time and allergen cost.^11^

In conclusion, the Oryum SPT device may be an acceptable alternative to the lancet in terms of sensitivity and reproducibility; furthermore, it may cause even less pain compared to the lancet. A less allergen expenditure and time-saving properties are the extra advantages of the test device. There are some differences among different devices and providers should consider these differences when deciding on a device. Not only the device used but the technique is also important when performing a SPT.

## Abbreviations

C: Clockwise
CC: Counter-clockwise
CV: Coefficient of variation
IgE: Immunoglobulin E
IQR: Interquartile range
P: Pressure
SD: Standard deviation
SPT: Skin prick test
W: Well
V: Vial

## Consent for publication

This study was approved by the local ethics committee (KA-180064) of the Hacettepe University Faculty of Medicine.

## Funding sources

There is no funding

## Author contributions

MK wrote the article, EK made the statistical analysis. ABB, OUS and UMS examined the patients and made the follow-ups. BES had primary responsibility for protocol development, out-come assessment and data analysis, and prepared the article with MK.

## Declaration of Competing Interest

Authors declare that there is no conflict of interest and no funding
